# Correlation Networks from Flows. The Case of Forced and Time-Dependent Advection-Diffusion Dynamics

**DOI:** 10.1371/journal.pone.0153703

**Published:** 2016-04-29

**Authors:** Liubov Tupikina, Nora Molkenthin, Cristóbal López, Emilio Hernández-García, Norbert Marwan, Jürgen Kurths

**Affiliations:** 1 Potsdam Institute for Climate Impact Research, P.O. Box 601203, 14412 Potsdam, Germany; 2 Humboldt Universität zu Berlin, 10099 Berlin, Germany; 3 Department of Physics, Technical University of Darmstadt, 64289 Darmstadt, Germany; 4 IFISC (CSIC-UIB), Instituto de Física Interdisciplinar y Sistemas Complejos, Campus Universitat de les Illes Balears, E-07122, Palma de Mallorca, Spain; Tianjin University, CHINA

## Abstract

Complex network theory provides an elegant and powerful framework to statistically investigate different types of systems such as society, brain or the structure of local and long-range dynamical interrelationships in the climate system. Network links in climate networks typically imply information, mass or energy exchange. However, the specific connection between oceanic or atmospheric flows and the climate network’s structure is still unclear. We propose a theoretical approach for verifying relations between the correlation matrix and the climate network measures, generalizing previous studies and overcoming the restriction to stationary flows. Our methods are developed for correlations of a scalar quantity (temperature, for example) which satisfies an advection-diffusion dynamics in the presence of forcing and dissipation. Our approach reveals that correlation networks are not sensitive to steady sources and sinks and the profound impact of the signal decay rate on the network topology. We illustrate our results with calculations of degree and clustering for a meandering flow resembling a geophysical ocean jet.

## Introduction

The network approach has become an essential tool in the study of complex systems [1–3], where networks are reconstructed from time series in order to uncover underlying dynamics [4–8]. Climate networks, i.e. those in which geographical nodes are linked when there is similar climatic dynamics on them (as measured by correlations, mutual information, etc.), have been thoroughly investigated in the last years in [9–14]. In the same context of geophysical systems, flow networks have also been introduced [15–18]. They are networks in which geographical nodes are linked when there is fluid transport from one location to another. Since correlations between different regions of a flow or geophysical system should be greatly influenced by the mass transport among them, it is natural to search for the relationship between these two types of networks, which will also help to understand the meaning of some of the teleconnections appearing in the climate network analysis. The works [19] and [20] are in this line, where networks were constructed from flow systems using a continuous analogue of the Pearson correlation. However these approaches have their limitations, mainly the restriction on the velocity fields to be constant in time. But the time-dependency plays an important role in real-world flows, for instance, all ocean currents vary over a large range of time scales [21–23].

In this paper we investigate general relationships between climate networks (specifically, networks built from correlations) and flow networks. In particular we develop a method for the analysis of time-dependent flows and demonstrate its potential for a specific model describing a meandering current. The quantity for which we compute spatial correlations is a scalar which is transported by the flow following an advection-diffusion dynamics. We can think on it as the ‘temperature’ of water in an ocean flow, but the formalism would apply to any transported quantity that could be considered ‘passive’ in some range of time scales. To avoid trivial homogenization, the scalar is forced by sources and sinks, which have both a spatially-dependent constant component and a time-varying stochastic part, and a decay process that prevents indefinite build-up, finally dissipating the input from the sources. By discretizing the system dynamics in space and time we obtain a linear recursive equation for the time-series of the scalar. We estimate the spatial correlation matrix from the time-series by averaging over various realizations of the noise. The correlation matrix can be thresholded, and interpreted as the adjacency matrix of the correlation network, which can then be analyzed using network measures which provides understanding of the formal relationship between the Lagrangian transport in the basic flows and the corresponding correlation network as used in climate networks.

The paper is organized as follows: First in the section Methods we introduce the tools for the construction of networks from general time-dependent flows, and describe our example meandering-jet model. The Results section describes the properties of our main formulae and illustrate them with the model flow. In the last section we discuss the main findings of the paper.

## Methods

We introduce an algorithm for the construction of correlation networks from the spatial distribution of a scalar (e.g. ‘temperature’) transported in a two-dimensional domain by an advection-diffusion equation (ADE) with additional forcing and decay terms:

$$\frac{\partial T(\vec{x},t)}{\partial t}=\kappa \Delta T\left(\vec{x},t\right)-\vec{v}\left(\vec{x},t\right)\cdot \nabla T\left(\vec{x},t\right)+F\left(\vec{x}\right)-bT\left(\vec{x},t\right)+\sqrt{D}\xi \left(\vec{x},t\right),$$ (1)

where *κ* is the diffusion coefficient, $\vec{v}\left(\vec{x},t\right)$ is the time-dependent bidimensional velocity field which we assume to be incompressible, $F(\vec{x})$ is the forcing, which describes time-independent sources and sinks, $\xi (\vec{x},t)$ is uncorrelated Gaussian white noise with zero mean and correlations $\langle \xi \left(\vec{x},t\right)\xi \left(\vec{y},t^{\prime }\right)\rangle =\delta \left(t-t^{\prime }\right)\delta \left(\vec{x}-\vec{y}\right)$. *D* is noise intensity and *b* is a damping parameter which sets the time-scale at which perturbations are dissipated in the system. We add decay and forcing to avoid convergence of the scalar distribution to a simple homogeneous equilibrium, and these processes are actually present in real geophysical flows [24].

### Discretised dynamics

The algorithm of network construction for a time-dependent velocity field requires first a discretisation of Eq (1). Let us consider first the simplified equation without forcing and decay:

$$\begin{array}{c} \frac{\partial T}{\partial t}=\kappa \Delta T-\vec{v}\left(\vec{x},t\right)\cdot \nabla T. \end{array}$$ (2)

We discretize Eq (2) using an Euler scheme for a regular *N* × *N*-lattice with spatial resolution Δ*x* and time-interval Δ*t*. The horizontal and vertical components of velocity field for the lattice point (*i*, *j*) at time step *k* = *t*/Δ*t* are $v_{ij}^{x}\left(k\right)$ and $v_{ij}^{y}\left(k\right)$. This gives:

$$\begin{array}{ccc} & & T_{ij}\left(k+1\right)=T_{ij}\left(k\right)-\\ & & \frac{\Delta t}{2\Delta x}\left(v_{ij}^{x}\left(k\right)T_{i+1j}\left(k\right)-v_{ij}^{x}\left(k\right)T_{i-1j}\left(k\right)+v_{ij}^{y}\left(k\right)T_{ij+1}\left(k\right)-v_{ij}^{y}\left(k\right)T_{ij-1}\left(k\right)\right)+\\ & & \frac{\kappa \Delta t}{\Delta x^{2}}\left(T_{ij+1}\left(k\right)+T_{ij-1}\left(k\right)+T_{i+1j}\left(k\right)+T_{i-1j}\left(k\right)-4T_{ij}\left(k\right)\right), \end{array}$$ (3)

where the node’s indices are *i*, *j* ∈ [1, *N*]. We use open boundary conditions. The discretisation parameters Δ*x* and Δ*t* should fulfill the Courant-Friedrichs-Lewy condition [25] for the stability of the discretisation scheme

$$\begin{array}{c} \frac{\kappa \Delta t}{\Delta x^{2}}<<1,\;\;\;\frac{max(v(x,t))\Delta t}{\Delta x}<<1. \end{array}$$

Eq (3) can be written in a matrix form in terms of the *N*^2^ × *N*^2^ one-step transformation matrix **P**(*k*) = **P**(*v*_*ij*_(*k*)) for time step *k* and the *N*^2^-dimensional state-vector *T*(*k*) of components ${\left(T(K)\right)}_{\vec{x}}\;=\;T_{ij\;}(k)$, with (*i*, *j*) the lattice coordinates of $\vec{x}$:

$$\begin{array}{c} T(k+1)=P(k)T(k). \end{array}$$ (4)

Iterating Eq (4) leads, for *k* ≥ *k*′, to

$$\begin{array}{c} T\left(k+1\right)=M_{kk^{\prime }}T\left(k^{\prime }\right), \end{array}$$ (5)

where

$$\begin{array}{c} M_{kk^{\prime }}=P\left(k\right)P\left(k-1\right)\ldots P\left(k^{\prime }+1\right)P\left(k^{\prime }\right) \end{array}$$ (6)

is the analogous to the transport matrix defining the flow networks in [16]. Here it is computed from a discretization of the ADE, whereas in other works [17, 18] it is computed by the Ulam method that involves the Lagrangian trajectories of particles, but the meaning is the same: it is the matrix that evolves in time the vector *T*(*k*).

Adding the decay term −*bT* to Eq (2):

$$\begin{array}{c} \frac{\partial T}{\partial t}=\kappa \Delta T-\vec{v}\left(\vec{x},t\right)\cdot \nabla T-bT \end{array}$$ (7)

does not pose technical difficulties, since the change of the variables $T\left(k\right)=e^{-b\Delta tk}\tilde{T}\left(k\right)$ reduces Eqs (7) to (2) for $\tilde{T}\left(k\right)$. Therefore the one-step solution Eq (4) becomes:

$$\begin{array}{c} T\left(k+1\right)=e^{-b\Delta t}P\left(k\right)T\left(k\right). \end{array}$$ (8)

Being a transport matrix, the eigenvalue with largest modulus of matrix **P**(*k*) is 1. The new one-step transformation e^−*b*Δ*t*^ **P**(*k*) will have eigenvalues which in modulus are smaller than 1, ensuring that perturbations become damped.

Reintroducing the forcing terms $F\left(\vec{x}\right)+\sqrt{D}\xi \left(\vec{x},t\right)$ from Eq (1) into the discretized framework Eq (3) can be done for example by integrating them with the Euler method. The one-step solution becomes

$$\begin{array}{c} T\left(k+1\right)=e^{-b\Delta t}P\left(k\right)T\left(k\right)+\Delta tF+s\epsilon \left(k\right). \end{array}$$ (9)

*F* is the time independent spatial forcing vector, and *ϵ*(*k*) is, at each time *k*, a vector of independent Gaussian random variables of zero mean and unit variance. These vectors are uncorrelated at different times. From the stochastic Euler method [26], the intensity of the discretized noise is $s=\sqrt{D\Delta t/\Delta x^{2}}$. Iteration of Eq (9) for (*k* + 1) time steps gives the time evolution of the scalar distribution vector:

$$\begin{array}{c} T\left(k+1\right)=G_{k0}\;T\left(0\right)+\Delta t\sum_{l=0}^{k}G_{kk+1-l}\;F+s\sum_{l=0}^{k}G_{kk+1-l}\;\epsilon \left(k-l\right)\;. \end{array}$$ (10)

We have introduced the propagation matrix, or propagator:

$$\begin{array}{c} G_{kk^{\prime }}\equiv e^{-b\Delta t}P\left(k\right)e^{-b\Delta t}P\left(k-1\right)\ldots e^{-b\Delta t}P\left(k^{\prime }\right)=e^{-(k+1-k^{\prime })b\Delta t}M_{kk^{\prime }}\;,\;k\geq k^{\prime }, \end{array}$$ (11)

and for notational convenience, we have defined

$$\begin{array}{c} G_{kk+1}\equiv I\;, \end{array}$$ (12)

the *N*^2^ × *N*^2^ identity matrix.

### Calculation of correlations

We are now able to compute the correlations associated to the time series generated by Eq (10). We consider the direct product matrix *T*(*k*)*T*(*k*)^†^ (the superindex † means transpose) whose matrix elements are products of the transported field at different spatial points $((k)T{\left(K\right)}^{†}){\;}_{\vec{x}\vec{y}}\;=\;T{\left(k\right)}_{\vec{x}}T{\left(k\right)}_{\vec{y}}^{†}$. We average it over realizations of the noise *ϵ*, operation which is denoted by 〈.〉. We also include in the same operation averaging over the initial condition *T*(0), for which we assume 〈*T*(0)〉 = 0. But we will see that in fact this assumption is irrelevant for our results, since the final expressions at long times lose dependence on the initial condition. Using $\langle \epsilon \left(k\right)\epsilon \left(k^{\prime }\right)\rangle =I\delta_{kk^{\prime }}$, we find:

$$\begin{array}{ccc} & & \left\langle T\left(k+1\right)T{\left(k+1\right)}^{†}\right\rangle =G_{k0}\left\langle T\left(0\right)T{\left(0\right)}^{†}\right\rangle G_{k0}^{†}+\\ & & {\left(\Delta t\right)}^{2}\sum_{l=0}^{k}\sum_{l^{\prime }=0}^{k}G_{kk+1-l}FF^{†}G_{kk+1-l^{\prime }}^{†}+s^{2}\sum_{l=0}^{k}G_{kk+1-l}G_{kk+1-l}^{†}\;. \end{array}$$ (13)

The first term in the r.h.s. of Eq (13) gives the evolution of the initial correlations. Because of the properties of the eigenvalues of **G**_*k*0_, this term will decrease with *k* and become negligible after a number *k* of steps such that the corresponding time *k*Δ*t* satisfies *bk*Δ*t* > >1. In the same limit, by averaging Eq (10), we see that

$$\begin{array}{c} \left\langle T\left(k+1\right)\right\rangle =\Delta t\sum_{l=0}^{k}G_{kk+1-l}F\;,\;b\Delta tk>>1\;, \end{array}$$ (14)

so that the second term in the r.h.s. of Eq (13) is 〈*T*(*k* + 1)〉〈*T*(*k* + 1)^†^〉. Combining these facts, we obtain for the spatial covariance of the transported scalar, if *bk*Δ*t* > >1:

$$\begin{array}{ccc} \text{Cov}(T(k)) & \equiv & \langle \left(T(k)-\langle T(k)\rangle \right){\left(T(k)-\langle T(k)\rangle \right)}^{†}\rangle \\ & = & s^{2}\sum_{l=0}^{k-1}G_{k-1k-l}G_{k-1k-l}^{†}\;. \end{array}$$ (15)

Expression Eq (15), with Eqs (11) and (12), gives the formal relationship between the correlations used to construct climate networks, obtained from the matrix Cov(*T*(*k*)), and the transport properties of the flow, which are contained in the flow-network matrix **M**_*kk*_′ and enter into Eq (15) via Eq (11).

### Network construction

From the covariance matrix we can calculate the Pearson correlation. In terms of the matrix elements of the covariance matrix, ${(\text{Cov(T(k)))}}_{\vec{x}\vec{y}}$, the matrix elements of the Pearson correlation matrix **C**(*k*) are:

$$\begin{array}{c} {\left(C(k)\right)}_{\vec{x}\vec{y}}=\frac{{\left(\text{Cov}(T(k))\right)}_{\vec{x}\vec{y}}}{\sqrt{{\left(\text{Cov}(T(k))\right)}_{\vec{x}\vec{x}}{\left(\text{Cov}(T(k))\right)}_{\vec{y}\vec{y}}}}\;. \end{array}$$ (16)

As standard for climate networks, we construct correlation networks from the symmetric and positive semi-definite matrices **C**(*k*). We threshold matrix **C**(*k*) to construct a binary adjacency matrix **A**(*k*):

$$\begin{array}{ccc} A{\left(k\right)}_{\vec{x}\vec{y}} & = & {1\;\;\;\text{if}\;|C\left(k\right)}_{\vec{x}\vec{y}}|⩾\gamma \\ A{\left(k\right)}_{\vec{x}\vec{y}} & = & {0\;\;\;\text{if}\;|C\left(k\right)}_{\vec{x}\vec{y}}|<\gamma \;. \end{array}$$ (17)

Within reasonable limits the value of the threshold value *γ* below which the correlations are set to zero does not significantly affect the result. The resulting thresholded matrix **A**(*k*) is the adjacency matrix of the correlation or climate network which is analyzed using network measures. In the following we will tune the threshold *γ* to obtain a network with a prescribed link density.

### A model flow

**Fig 1:**
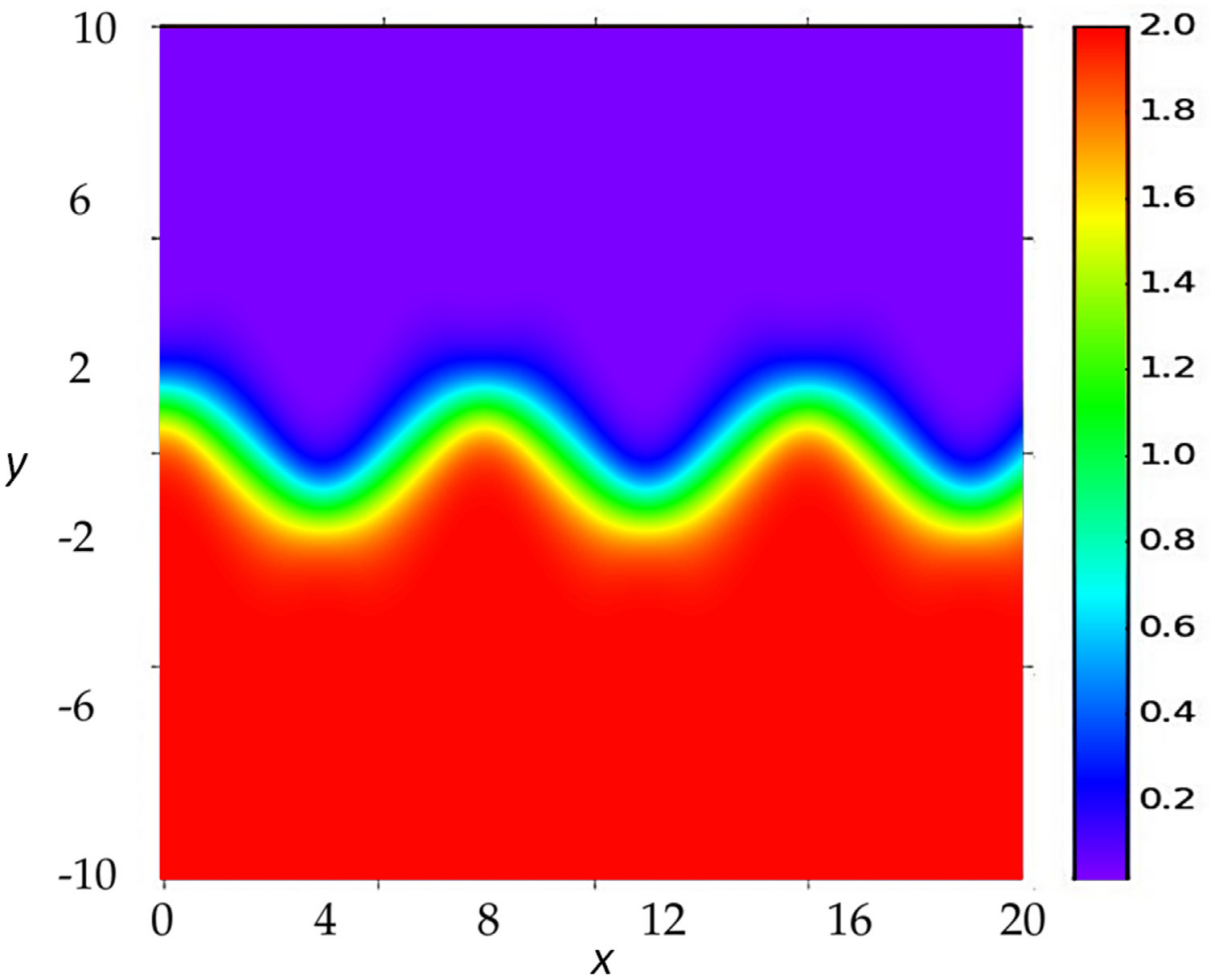
The streamfunction for the velocity field of the meandering flow. It describes a jet flowing from left to right, more intense in the central meandering core. The streamfunction is plotted here for *ν* = 0, and it is the same as for any other value of *ν* if *t* = 0 or a multiple of the flow period. Other parameters are given in the text.

To illustrate the use of the formulae derived above, we choose a *meandering flow* model [27, 28] to construct the flow-networks. It resembles the simplified velocity structure present in ocean currents such as the Gulf Stream or the Kuro-Shio. Following [29] the streamfunction is given by:

$$\begin{array}{c} \Psi \left(x,y,t\right)=1-\tanh\left[\frac{y-B\left(t\right)\cos\left(m(x-ct)\right)}{{\left[1+m^{2}B{\left(t\right)}^{2}\sin^{2}\left(m(x-ct)\right)\right]}^{\frac{1}{2}}}\right], \end{array}$$ (18)

where *m* is a wave (meander) number which we set to 2π/*L*_*x*_, *L*_*x*_ = 7.5 and *B*(*t*) is the wave amplitude, given by *B*(*t*) = *B*_0_ + *ν* cos(*ωt* + *θ*). A snapshot of the streamfunction Eq (18) is plotted in Fig 1. It describes a jet flowing towards the positive *x* direction, more intense in the central core region, and meandering in the *y* direction. A meandering flow is well-studied flow model [30, 31]. Moreover, regions of the velocity field, denoted by Eq (18), contain flows with more simple structure. Altogether this makes a meandering flow a suitable model to test a novel flow networks method. We fix parameters at *B*_0_ = 1.2, *c* = 0, *ω* = 0.4, *θ* = *π*/2, and compare results for the static, *ν* = 0, or oscillating in amplitude, *ν* = 0.7, meander. In the first case particle motion in the flow is integrable whereas in the second chaotic motions arise [28, 29]. From Ψ(*x*, *y*, *t*) the velocity field $\vec{v}=\left(v^{x},v^{y}\right)$ is calculated as:

$$\begin{array}{c} v^{x}\left(x,y,t\right)=-\frac{\partial \Psi (x,y,t)}{\partial y}\;\;,\;\;v^{y}\left(x,y,t\right)=\frac{\partial \Psi (x,y,t)}{\partial x}\;. \end{array}$$ (19)

## Results

In the case without advection (or advection with a constant and homogeneous velocity field $\vec{v}\left(x,y,t\right)={\vec{v}}_{0}$) Eq (1) can be solved exactly and the Pearson correlation computed. The resulting network is a fully homogeneous graph in which every node is linked with all neighbor nodes within a correlation length given by $\sqrt{\kappa /b}$. In the presence of non-homogeneous advection, the network becomes inhomogeneous with properties determined by Eq (15) which encodes, via the propagator **G**_*kk*_′, a non-trivial interplay between advection, diffusion and decay. Here are some implications of our main formula Eq (15):

- In the framework of the linear ADE dynamics we are using here, a time-independent spatial forcing $F(\vec{x})$ has no influence on the covariance matrix, as it is constructed from anomalies with respect to the mean. In the same way, white noise intensity *s* or *D* disappears when normalizing the covariance to obtain the Pearson correlation coefficient of Eq (16). Thus correlation networks become independent from the forcing terms present in the linear ADE Eq (1) (although these terms need to be present to sustain the fluctuations from which correlations are computed). The choice of the white noise in Eq (1) was motivated by [32], where the effect of the random weather excitation on the ocean dynamics is represented by the white noise.
- For flow networks constructed from the transport matrix **M**_*kk*_′ (or **G**_*kk*_′), nodes are connected if there is physical transport between them. For networks constructed from the correlation (16), instead, the presence of the product of two propagators, $G_{k-1k-l}G_{k-1k-l}^{†}$, in each term of the sum in Eq (15) implies that correlations between two nodes will be non-vanishing only if they receive simultaneously (at time *k*) the effect of fluctuations originated at the same source (at time *k* − *l*). This cannot happen only by advection, because Lagrangian trajectories are predetermined by deterministic flow model. Diffusion is needed to spread stochastic perturbations and let them to affect different sites. Thus, links between nodes in correlation networks constructed from transported quantities will not represent direct physical transport between them, but the susceptibility for them to be reached by perturbations transported (by advection and diffusion) from the same origin (and within a time *b*^−1^ from its birth, because of the exponentially decaying temporal factor in **G**_*kk*_′).
- Even if for large integration time *k* Eq (15) involves a large number of terms in the sum, they decrease fast in magnitude, and actually only the ones with *l* such that *b*(*k* − *l*)Δ*t* < 1 make a relevant contribution to the covariance or Pearson correlation at time *k*.
- Cov(*T*(*k*)) is a time-dependent matrix, as it depends on **G**_*kk*_′ and thus on **P**(*k*), which inherits the time-dependence on the velocity field $\vec{v}\left(\vec{x},t\right)$. Because of the temporal averaging implicit in Eq (15), temporal scales of the velocity field faster than the time scale *b*^−1^ will be averaged out from Cov(*T*(*k*)), but slower time-dependencies will remain and the resulting correlation network will be a temporal network [4].

We illustrate these general results with numerical computations of correlations via Eqs (15) and (16) for the ADE dynamics with the meandering model flow, and construction of the associated networks. We consider the domain *x* ∈ [0, 20], *y* ∈ [−10, 10] with open boundary conditions and discretize it in *N* × *N* = 120 × 120 nodes, so that Δ*x* ≈ 0.167. Time step is Δ*t* = 0.2. We nominally take the diffusion coefficient *κ* = 0.02, but the numerical diffusion [25] introduced by the discretization Eq (3) is larger, *κ*′ ≈ Δ*x*^2^/Δ*t* = 0.139. We consider two different regimes for the damping: *b* = 1 and *b* = 0.05, corresponding to lifetimes of the perturbations much shorter (*b*^−1^ = 1) than the time scales of the flow (as given by 2π/*ω* ≈ 15.7), or longer (*b*^−1^ = 20). For the flow all parameters are fixed as mentioned above, except the one giving the temporal modulation of the meander amplitude: *ν* = 0, representing a steady flow or *ν* = 0.7, giving a time-dependent flow.

**Fig 2:**
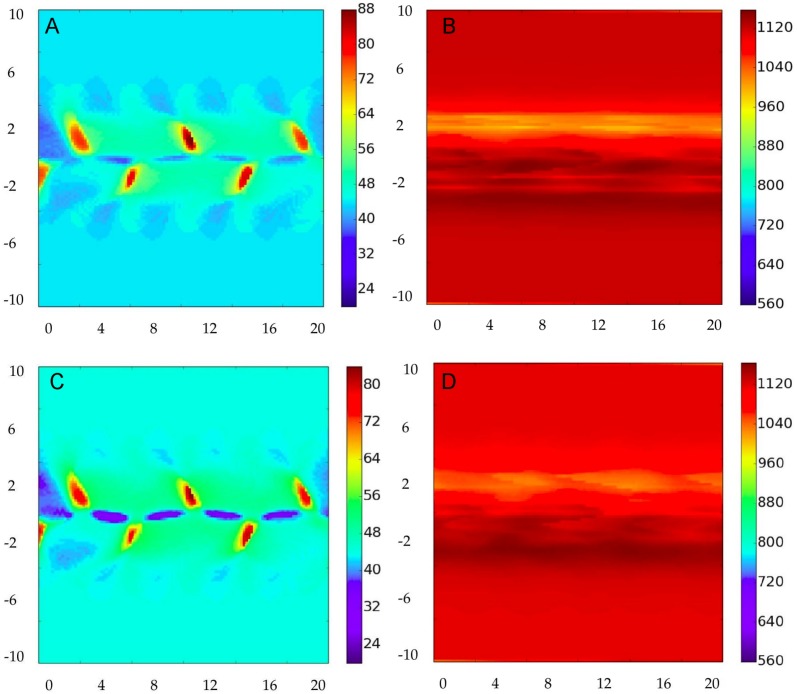
Node degree centrality for the correlation networks constructed for different flows and decay rates. The direction *x* is horizontal and *y* is the vertical. Panels A and B display the case of the static flow, *ν* = 0. C and D are for the amplitude-changing case, *ν* = 0.7. The network for the dynamic case is plotted at a time after *t* = 0 multiple of the flow period. Then, for all panels the streamfunction at the time plotted is the one shown in Fig 1. Panels A and C are for the fast decay case *b* = 1, and B and D are for the slow decay, *b* = 0.05, of the transported substance. Other parameters as stated in the text.

**Fig 3:**
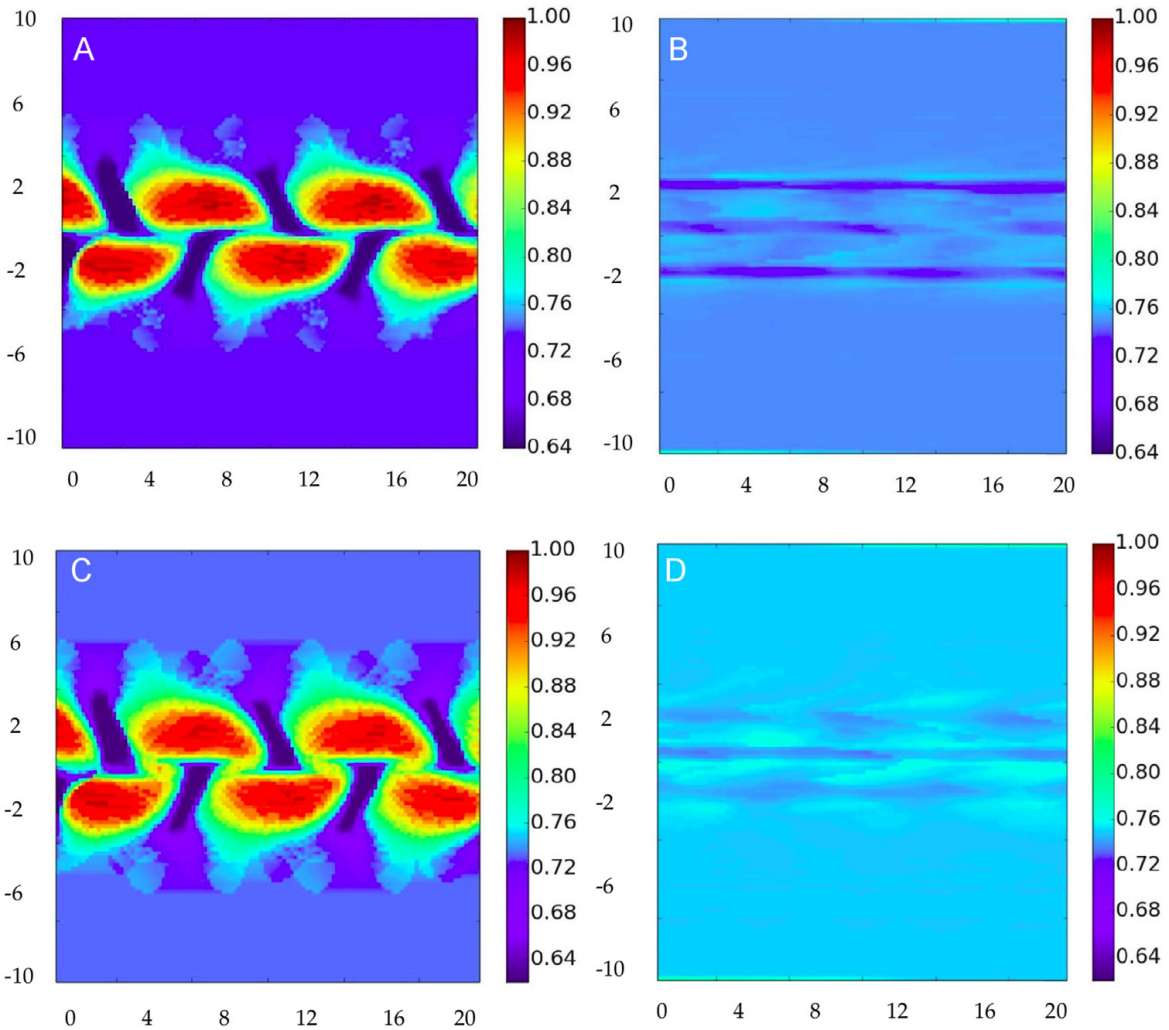
Node clustering coefficient for the correlation networks constructed for different flows and decay rates. Panels are for the same parameters as in Fig 2.

The network adjacency matrix **A**(*k*) is constructed from Eqs (15), (16) and (17). We find that using in the sum of Eq (15) a number of terms *k* = 314 for *b* = 1 and *k* = 942 for *b* = 0.05 (which satisfy the condition *bk*Δ*t* > >1) is sufficient to pass the spin-up period in which the initial correlations (the first term in the right-hand-side of Eq 13) are still important, and to reach the asymptotic statistical regime. When *ν* = 0 the flow is static, with streamfunction plotted in Fig 1, and then the network constructed from **A**(*k*) is also static. When *ν* ≠ 0 the flow, and then the correlations and the network, is periodic with period 2π/*ω*. For the values used for *k*, the times *k*Δ*t* correspond to exactly 4 or 12 periods after time *t* = 0 so that at these instants the streamfunction is also the one plotted in Fig 1. To highlight the spatial structures in the network we fix the threshold *γ* such that the node density is 0.075 for the cases with *b* = 0.05, and 0.003 for *b* = 1. Because of the different values we cannot directly compare the absolute values of the network metrics computed at different *b*. But we will be only interested in the spatial patterns. We have checked that, although details of the degree and clustering distributions vary, changing the link density in a factor of two does not alter the location of the regions of high and low values of degree and clustering with respect to the ones in Figs 2 and 3.

To analyze the network structure we calculate standard network measures [1, 33, 34]: *node degree centrality*, which is the number of links adjacent to the node, and *node clustering coefficient*, which is the fraction of triangles actually present through that node with respect to the possible ones, given their neighbors. The degree of the nodes in the network is plotted in Fig 2 for the four combination of parameters involving *ν* = 0, 0.7 and *b* = 1, 0.05. Fig 3 displays the corresponding clustering values.

In the static case (*ν* = 0, panels A and B of Figs 2 and 3) the streamfunction, given in Eq (18) is constant in time, and plotted in Fig 1. As expected from Eq (15) and the discussion above, regions of high degree are not precisely associated with strong currents. Nevertheless, when damping rate is fast (*b* = 1, Fig 2A) the general spatial structure of the degree reflects the meandering shape of the flow. The similarity is stronger between flow and clustering plots (Fig 3A): patches of strong clustering follow the meander structure, with high clustering usually associated to zones of low degree, and viceversa.

The situation completely changes for *b* = 0.05 (Figs 2B and 3B). Here both degree and clustering become nearly homogeneous, with only some weak structure elongated on the horizontal *x* direction. The reason is that now many terms corresponding to different times contribute to the sum in Eq (15), averaging the resulting correlations that loose spatial structure.

If we turn on now the temporal dependence of the flow, *ν* = 0.7, little changes are seen. For the case *b* = 1 (Figs 2C and 3C) this is easy to interpret, since as discussed above only a few terms in the sum in Eq (15), the ones with (*k* − *l*)*b*Δ*t* < 1, contribute. For them the flow stays essentially unchanged (the time scale for changes in the flow is 2π/*ω* ≈ 15.7 ≫ *b*^−1^ = 1). Thus the results should be nearly equivalent to the static case. In fact only small increases in degree in the central parts and decreases of degree at the maxima are seen in Fig 2C with respect to the static case Fig 2A. Despite the long-time transport properties are rather different in the static and time-dependent case (in particular Lagrangian transport is chaotic at *ν* = 0.7 [29]) a large damping *b* restricts the correlations to be influenced only by the short term dynamics, which is similar to the static case.

Making the decay rate slower (*b* = 0.05, Figs 2D and 3D) in this dynamic case for *ν* = 0.7 has also the consequence of homogenizing the spatial structure, in a manner similar to that of the static case. The structure is here slightly more homogeneous than for *ν* = 0, because of the additional mixing associated to the chaotic dynamics.

## Discussion and outlook

The results shown above close a gap in the theoretical understanding of the relationship between networks constructed from correlation functions, as usually done for climate networks, and the underlying dynamics of the fluid transport.

A first observation is that, when the Pearson correlation is used to establish links between nodes, correlation networks are not sensitive to steady sources and sinks of the transported substances. Also the normalization in Eq (16) eliminates the dependence on fluctuation intensity. As a consequence in geophysical contexts, one cannot look into climate networks for information about these processes. Note that this implication is strictly valid only for the linear ADE dynamics in Eq (1) and will not apply to dynamics involving nonlinear processes (plankton dynamics, vorticity, …). Also, it may not hold when nonlinear measures of statistical dependence, such as mutual information, information transfer [14, 35] or event synchronization [11] replace the correlation function.

Another important point, evident from Eq (15), is that the relationship between the correlation network, constructed from **C**(*k*) and the underlying flow transport network (characterized by **M**_*kk*_′ or **G**_*kk*_′) is not direct, since the correlation expression involves a sum over time, and each term involves the product of two propagators, meaning that correlated nodes are not the ones connected by the flow, but the ones affected within a time *b*^−1^ by perturbations coming from a common origin. It is straightforward to repeat the calculations for the case in which a colored noise correlation is used for *ϵ*(*k*). The result is that correlated nodes are the ones affected by perturbations coming from locations within the same correlation length and time of the noise. In consequence, patterns of degree or of other network measures are related to flow patterns in a rather indirect way, as Figs 2 and 3 confirm. Note that this result relies strongly on considering the *equal-time correlation*. In cases in which a *time-lagged correlation* is used [9, 19, 36], the resulting network would be more associated to fluid transport occurring between nodes during the selected temporal lag. Also, our analysis in this paper is restricted to the ADE dynamics implemented by Eq (1), which considers only material transport. Our conclusions may not apply to climate networks constructed from variables involving wave propagation (Kelvin, Rossby, …), such as sea surface height or geopotential [37].

From the numerical results presented here it is seen that one of the parameters having the largest impact on the network topology, in fact more than the flow geometry or temporal variability, is the characteristic time scale of perturbation damping (here represented by the decay rate *b*). This important parameter would then have to be taken into account when investigating the structure of climate networks constructed from observed or analyzed data.

In summary we have elucidated, in the context of ADE dynamics, general relationships between correlation and flow networks, overcoming some restrictions of previous approaches, [19]. Moreover, flow networks are further applicable, for instance, to study changes in flow behavior [38, 39]. All in all, the methods above can, in principle, be applied in other contexts, in which temporal networks [4, 40, 41] are used in order to study transport process, so the present framework can be useful to investigate different complex systems.
